# Effect of Exercise on Liver Function and Insulin Resistance Markers in Patients with Non-Alcoholic Fatty Liver Disease: A Systematic Review and Meta-Analysis of Randomized Controlled Trials

**DOI:** 10.3390/jcm12083011

**Published:** 2023-04-20

**Authors:** Keyvan Hejazi, Daniel Hackett

**Affiliations:** 1 Department of Physical Education and Sport Sciences, Hakim Sabzevari University, Sabzevar 9617976487, Iran; 2 Discipline of Exercise & Sport Science, Health and Performance Faculty Research Group, Faculty of Medicine and Health, Sydney School of Health Sciences, The University of Sydney, Camperdown, NSW 2006, Australia

**Keywords:** non-alcoholic fatty liver disease, insulin resistance, exercise training, liver enzymes, meta-analysis

## Abstract

Background: Structured exercise as part of lifestyle modification plays an important role in the improvement of non-alcoholic fatty liver disease (NAFLD); however, its effectiveness has been shown to vary. This systematic review with meta-analysis investigated the effects of exercise on liver function and insulin resistance markers in patients with NAFLD. Methods: Six electronic databases were searched using terms related to exercise and NAFLD up to March 2022. Data were analyzed using a random-effects model to estimate the standardized mean difference (SMD) and 95% confidence interval. Results: The systematic search identified 2583 articles, of which a total of 26 studies met the inclusion criteria and were eligible. Exercise training had a moderate effect on reducing ALT (SMD: −0.59, *p* = 0.01) and small effects on reducing AST (SMD: −0.40, *p* = 0.01) and insulin (SMD: −0.43, *p* = 0.02). Significant reductions in ALT were found following aerobic training (SMD: −0.63, *p* < 0.01) and resistance training (SMD: −0.45, *p* < 0.001). Moreover, reductions in AST were found following resistance training (SMD: −0.54, *p* = 0.001), but not after aerobic training and combined training. However, reductions in insulin were found following aerobic training (SMD: −0.55, *p* = 0.03). Exercise interventions for <12 weeks compared to ≥12 weeks were more effective in reducing FBG and HOMA-IR, while interventions for ≥12 weeks compared to <12 weeks were more effective in reducing ALT and AST levels. Conclusions: Our findings support the effectiveness of exercise in improving liver function markers but not in blood glucose control in NAFLD patients. Additional studies are needed to determine the exercise prescription to maximize health in these patients.

## 1. Introduction

Non-alcoholic fatty liver disease (NAFLD) is a prevalent hepatic disorder that is accompanied by the concentration of lipids in hepatocytes and usually arises when the concentration of fats in the liver exceeds 5% of the organ weight [1,2]. The disorder ranges from steatosis and non-alcoholic steatohepatitis (NASH) to advanced fibrosis and cirrhosis [3,4]. NAFLD is among the most prevalent forms of chronic hepatic diseases in the world and is the main reason for adults visiting hepatology clinics [5,6]. The disease affects almost 25% of the world population [7] and 23% of European citizens [8].

Over the past few years, NAFLD has attracted a lot of attention as a risk factor for the development of insulin resistance (IR) and type 2 diabetes mellitus (T2DM) [9,10]. Studies indicate that NAFLD is associated with obesity, insulin resistance, T2DM, and dyslipidemia, and its prevalence throughout the world appears to be increasing [11]. Currently, there is no pharmacological treatment for NAFLD, therefore the initial management focuses on lifestyle and dietary changes [12,13]. Engaging in adequate levels of physical activity plays a protective role against the emergence of T2DM, central (abdominal) obesity, and other risk factors related to NAFLD (including high blood pressure and dyslipidemia) [14]. Exercise interventions in the absence of dietary changes (with or without weight loss) have been shown to improve clinical markers in patients with NAFLD [12]. This may include improvement in markers such as intrahepatic lipids (IHLs), levels of alanine aminotransferase (ALT) and aspartate aminotransferase (AST), and insulin resistance.

The European Association for the Study of the Liver (EASL) recommends 150–200 min per week of medium-intensity exercise for patients with NAFLD [15]. The effects of different exercise modalities in patients with NAFLD have produced conflicting results [16,17,18,19]. Two systematic reviews with meta-analyses in patients with NAFLD found that aerobic training, resistance training, and combined exercise training (aerobic training + resistance training) did not improve hepatic enzymes including ALT and AST [16,17]. A meta-analysis conducted by Xiong et al. including 1250 NAFLD patients showed that aerobic exercise led to reductions in more metabolic and liver function markers compared to resistance training and high-intensity [18]. Zhou et al. conducted a meta-analysis on 1627 NAFLD patients and found that combined exercise training produced the most favorable changes in total cholesterol (TC), ALT, and AST, compared to that with performing a single exercise modality (i.e., aerobic training, resistance training, or HIIT). However, it should be noted that for the previous meta-analysis, no significant differences between the exercise modalities were found for the hepatic enzymes [19].

Therefore, the most effective exercise modality for improvements in clinical markers of NAFLD is yet to be determined. Moreover, there are conflicting results concerning the effect of exercise on specific hepatic enzymes and their roles in the prevention and treatment of NAFLD [16,17,18,19]. To provide practitioners with the optimal exercise therapeutic approach for the management of NAFLD requires an up-to-date synthesis of evidence from randomized controlled trials (RCTs). Thus, the purpose of this study was to assess the effect of exercise on liver enzymes and insulin resistance markers in NAFLD patients via a systematic review and meta-analysis. A second aim was to examine whether the effects of an exercise intervention on liver enzymes and insulin resistance markers were influenced by exercise modality, intervention duration, and body mass index (BMI) status.

## 2. Materials and Methods

### 2.1. Search Strategy

This systematic review and meta-analysis was conducted according to the recommendations outlined in the Preferred Reporting Items for Systematic Reviews and Meta-Analyses (PRISMA) statement (Supplementary Table S1) [20]. A detailed search was performed using six databases, including Web of Science, Embase, Cochrane, PubMed, Google Scholar, and Scopus. The search was conducted from the earliest record up to 5 October 2021, with an updated search up to and including 3 March 2022. The search strategy combined the terms: “liver steatosis”, “non-alcoholic fatty liver disease”, “NAFLD”, “randomized controlled trial”, “fatty liver”, “liver”, “hepatic”, “NASH”, “aminotransferase”, ‘‘liver enzymes’’, ‘‘AST’’, ‘‘ALT’’, ‘‘FBG’’, ‘‘fasting insulin’’, ‘‘HOMA-IR’’, “strength training”, “physical activity”, “combined training”, “exercise training”, “walking”, “aerobic training”, “resistance training”, “circuit training”, “circuit’’, ‘‘weight training”, “interval training”, “chronic training”, “ lifestyle activity”, “home training”, ‘‘water exercise’’, and ‘‘tai chi’’. All relevant studies and other available systematic reviews (including meta-analyses) and the reference lists of the identified articles were hand searched for additional eligible studies.

Where possible, searches were limited to studies involving human participants and published in the English language. Titles and abstracts of retrieved articles were individually evaluated by two reviewers (K.H. and D.H.) to assess their eligibility for review and meta-analysis. The reviewers were not blinded to the studies’ authors, institutions, or journals of publication. When abstracts did not provide sufficient information to examine study eligibility, the full-text was retrieved for evaluation. Duplicate publications were identified by comparing author names, treatment comparisons, publication dates, sample sizes, intervention, and outcomes.

### 2.2. Eligibility Criteria

Articles were eligible for inclusion if they met the following criteria: randomized controlled trials; peer-reviewed articles published in English; an exercise intervention of ≥4 weeks duration; adults (≥18 years) diagnosed with NAFLD or NASH; and reported post-intervention change in liver enzymes and insulin resistance markers. These markers specifically included the following: enzymes of the liver (e.g., ALT, AST), fasting blood glucose (FBG), insulin, and homeostatic model assessment for insulin resistance (HOMA-IR). The studies also required participants to be physically inactive (<150 min per week of moderate intensity aerobic exercise or <75 min per week of vigorous aerobic exercise) and no participation in an exercise program within the previous 6 months. Articles were deemed ineligible if only the abstract was available; did not involve humans (i.e., animal studies); and cohorts had secondary causes of fatty liver such as alcohol, hepatitis viruses, or medication.

### 2.3. Data Extraction

The information extracted comprised publication details (such as first author’s name, year of publication, and country), details of the study (i.e., sample size for the intervention and control group and health status), participants’ characteristics (i.e., mean age of participants), description of the intervention (i.e., type of exercise, intervention period, frequency, intensity, duration, sets, and repetition). Mean and standard deviation (SD) of the liver enzymes (ALT and AST) and insulin resistance marker (FBG and HOMA-IR) values at baseline, post-intervention, and/or changes between baseline and post-intervention were noted. If standard errors were reported, these values were converted to SD [21]. Any data presented in graphs were extracted using web-based software (GetData Graph Digitizer) [22]. If data was unavailable or could not be extracted from figures, the study’s corresponding author was emailed to request the data. All data were extracted by two authors (K.H. and D.H.).

### 2.4. Study Quality

The methodological quality of the studies were examined independently based on a fifteen-point scale, Tool for the Assessment of Study Quality and Reporting in Exercise (TESTEX), which is a validated tool for evaluating the quality (five points maximum) and reporting (10 points maximum) of exercise training studies [23]. Two authors (K.H. and D.H.) carried out this evaluation.

### 2.5. Statistical Analysis

All meta-analyses were conducted with Review Manager 5.3 (The Nordic Cochrane Centre, Copenhagen, Denmark). Effect size (ES) values were expressed as standardized mean difference (SMD) and 95% confidence interval (CI). The SMD was calculated using the formula shown below:

(1) $$\mathrm{SMD}=\frac{\mathrm{mean}\;\left(\mathrm{DInt}\right)-\mathrm{mean}\;\left(\mathrm{DCtr}\right)}{\mathrm{SD}\;\mathrm{pooled}}$$

where DInt and DCtr are the post–pre differences in the exercise intervention and control groups, respectively, and SDpooled is the pooled standard deviation [24]. The ESs were interpreted by Cochrane guidelines, with SMD = 0.20–0.49 indicating a small ES, SMD = 0.50–0.79 indicating a medium ES, and SMD > 0.80 indicating a large ES [25].

When a study involved a control group and more than one exercise group, ESs were calculated for each exercise group and the sample size of the control group was divided by the number of exercise groups. Between-study variability was examined for heterogeneity using the I^2^ statistic for quantifying inconsistency. Heterogeneity thresholds included I^2^ = 25% (low), I^2^ = 50% (moderate), and I^2^ = 75% (high) [21]. A conservative random-effects model of meta-analysis was applied to the pooled data. Significance was set as *p* < 0.05. Subgroup analyses were employed to recognize potential causes of heterogeneity among the studies. Exercise training modality (i.e., aerobic, resistance, and combined aerobic + resistance); exercise duration (i.e., <12 weeks versus ≥12 weeks); and body mass index (BMI) (overweight: 25.0–29.9 kg/m^2^ versus obese: ≥30 kg/m^2^), were considered as the predefined sources of heterogeneity. Only one study that met the inclusion criteria investigated the effects of interval training on the outcomes of interest [26]. Therefore, it was decided that this study would not be included in any analysis. Publication bias was examined visually via funnel plots and statistically using Egger’s test (*p* < 0.05) [27].

## 3. Results

### 3.1. Study Selection

**Figure 1:**
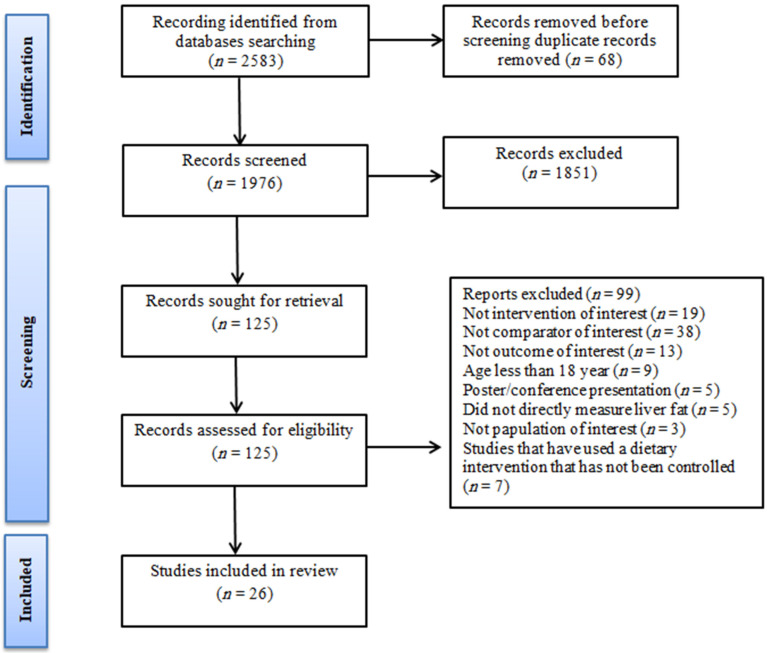
PRISMA flow diagram of the literature search.

The initial search identified 2583 records. After removing duplicates and following title/abstract screening, 125 studies were included for full-text review. Of those 125 studies, 26 studies met the eligibility criteria. A flow diagram of the study search and selection, including reasons for exclusion, is shown in Figure 1.

### 3.2. Study and Participant Characteristics 

Relevant characteristics of the included studies are presented in Table 1. Studies were carried out in Egypt (one study), India (one study), Brazil (one study), China (two studies), USA (two studies), Japan (one study), Australia (three studies), the UK (four studies), and Iran (11 studies).

There were 1316 participants included in this review (intervention groups: 800 participants; control groups: 516 participants). Of the 26 included studies, 10 studies exclusively recruited male subjects (*n* = 401) [28,29,30,31,32,33,34,35,36,37], 4 studies exclusively recruited female subjects (*n* = 303) [38,39,40,41], and 12 studies recruited both (male + female) (*n* = 507) [26,42,43,44,45,46,47,48,49,50,51,52].The mean age of subjects ranged from 18 to 66 years. The average BMI was 30.81 kg/m^2^. Furthermore, 11 studies involved overweight subjects (BMI of ≥25–29.9 kg/m^2^) [29,31,32,33,35,39,41,45,47,51,52], 14 studies involved obese subjects (BMI of ≥30 kg/m^2^) [30,34,36,37,38,40,42,43,44,46,48,49,50,53], and only one study did not report BMI [28].

**Table 1:** Characteristics of the included studies.

			Participants				
Author, Year	Country	Disease	Ex (CON)	BMI	Gender (M/F)	Age	Outcome Assumed
Abdelbasset et al., 2019 [26]	Egypt	NAFLD	16 (16)	36.1	M F	54.8	ALT, HBA1c, FBG, HOMA-IR
Cuthbertson et al., 2016 [42]	UK	NAFLD	30 (20)	30.15	M F	51	ALT, AST, FBG, HOMA-IR, Insulin
Eckard et al., 2013 [37]	USA	NAFLD	9 (11)	33.3	M	51.5	ALT, AST, FBG, HOMA-IR, Insulin
George et al., 2008 [36]	Australia	NAFLD	109 (34)	31.7	M	48.12	ALT, AST, FBG, HOMA-IR, Insulin
Hallsworth et al., 2011 [43]	UK	NAFLD	11 (8)	32.3	M F	57	ALT, FBG, HOMA-IR, Insulin
Hatami et al., 2016 [35]	Iran	NAFLD	16 (16)	28	M	32.93	ALT, AST
Hoseini et al., 2020 [38]	Iran	NAFLD	10 (10)	34.11	F	62.3	ALT, AST, FBG, HOMA-IR, Insulin
Houghton et al., 2017 [44]	Australia	NAFLD	12 (12)	33.00	M F	52.00	ALT, AST, FBG, HOMA-IR
Javanmardi Fard et al., 2015 [45]	Iran	NAFLD	30 (30)	28.65	M F	19	ALT, AST
Keating et al., 2015 [46]	Australia	NAFLD	36 (12)	33.42	M F	43.6	ALT, AST, FBG, Insulin
Khaoshbaten et al., 2013 [47]	Iran	NAFLD	45 (45)	29.2	M F	37.6	ALT, AST, FBG
Mohammadi et al., 2019 [34]	Iran	NAFLD	10 (10)	32.58	M	37.2	ALT, AST
Moradi Kelardeh et al., 2017 [33]	Iran	NAFLD	12 (12)	29.27	M	38.24	ALT, AST
Moradi Kelardeh et al., 2020 [39]	Iran	NAFLD	12 (11)	27.36	F	65.27	ALT, AST
Nourian et al., 2020 [48]	Iran	NAFLD	36 (33)	32.19	M F	48.84	ALT, AST
Pugh et al., 2014 [49]	UK	NAFLD	34 (20)	30.5	M F	47.5	ALT, AST, FBG, HOMA-IR, Insulin
Rezende et al., 2016 [40]	Brazil	NAFLD	19 (21)	33.05	F	55.3	ALT, AST, FBG, HOMA-IR, Insulin
Sadeghi et al., 2019 [32]	Iran	NAFLD	11 (11)	26.96	M	40.8	ALT, AST
Shamsoddini et al., 2015 [31]	Iran	NAFLD	20 (10)	28.96	M	43.8	ALT, AST, HOMA-IR
Shojaee-Moradie et al., 2016 [30]	UK	NAFLD	15 (12)	31.65	M	52.6	ALT, AST, FBG, HOMA-IR, Insulin
Sreenivasa Baba et al., 2006 [29]	India	NAFLD	44 (15)	27.1	M	38.7	ALT, AST
Sullivan et al., 2012 [50]	USA	NAFLD	12 (6)	38.1	M F	48.05	ALT
Takahashi et al., 2015 [51]	Japan	NAFLD	31 (22)	28.35	M F	53.45	ALT, AST, FBG, HOMA-IR, Insulin
Valizadeh et al., 2011 [28]	Iran	NAFLD	12 (12)	NR	M	32.5	ALT, AST
Yao et al., 2018 [52]	China	NAFLD	60 (31)	26.26	M F	58.38	ALT, FBG, Insulin
Zhang et al., 2016 [41]	China	NAFLD	146 (74)	28.0	F	53.9	ALT, AST, FBG

Note: The control group received no training. Abbreviations: M, male; F, female; NAFLD, nonalcoholic fatty liver disease; FBG, fasting blood glucose; ALT, alanine aminotransferase; AST, aspartate aminotransferase; HOMA-IR, homeostatic model assessment for insulin resistance; n.r., not rated.

### 3.3. Intervention Characteristics

Exercise intervention characteristics are summarized in Table 2. The duration of exercise interventions ranged from 4 to 48 weeks, with the duration of individual exercise sessions ranging from 20 to 200 min. Frequency of exercise training ranged between two and seven sessions per week.

Fifteen studies directly examined the effects of aerobic training [28,29,30,36,37,38,40,41,42,45,46,47,48,49,50], six directly examined the effects of resistance training [32,33,34,39,43,51], two studies directly investigated the effects of aerobic training versus resistance training [31,52], two studies directly investigated the effects of combined training (i.e., aerobic and resistance training) during sessions [35,44], and one study directly investigated the effects of interval training [26].

### 3.4. Effect of Exercise on Hepatic Enzyme Parameters

#### 3.4.1. ALT Levels

**Figure 2:**
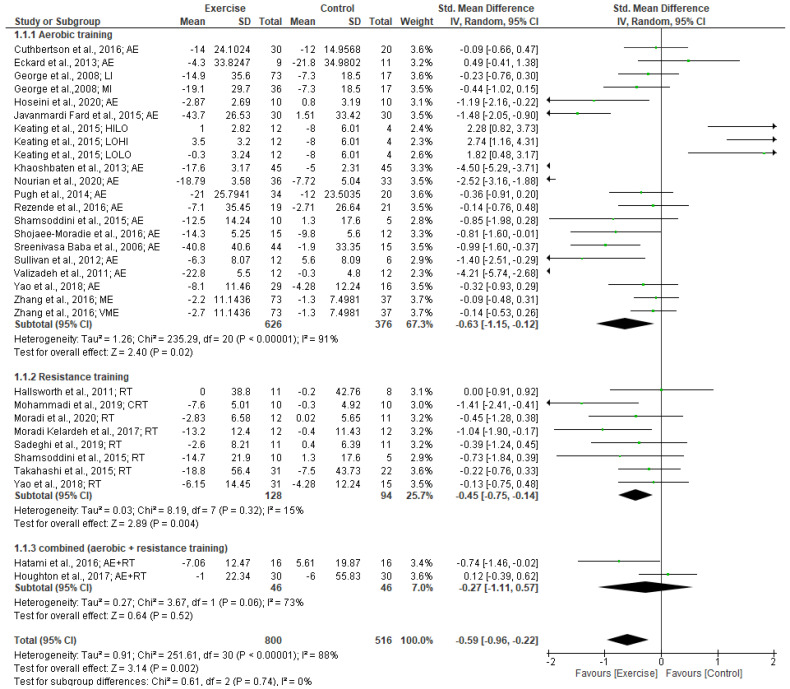
Forest plot showing study precision against the mean difference effect estimate with 95% confidence interval for ALT [28,29,30,31,32,33,34,35,36,37,38,39,40,41,42,43,44,45,46,47,48,49,50,51,52].

Our meta-analysis of 25 studies revealed a significantly moderate effect of exercise training in reducing ALT levels (SMD: −0.59 [95% CI, −0.96 to −0.22], *p* = 0.002; (I^2^ = 88%, *p* < 0.001). The subgroup analysis of exercise type revealed that aerobic training had a significantly moderate effect on ALT levels (SMD: −0.63 [95% CI, −1.15 to −0.12], *p* = 0.02; I^2^ = 91%, *p* < 0.001). Resistance training had a significantly small effect on ALT levels (SMD: −0.45 [95% CI, −0.75 to −0.14], *p* < 0.004; I^2^ = 15%, *p* < 0.32). There was no significant effect of combined exercise training (aerobic + resistance) on AST levels (see Forest plot in Figure 2).

A subgroup analysis of exercise intervention duration indicated that a duration of ≥12 weeks significantly reduced ALT levels. Moderate effects were found for exercise training for ≥12 weeks (SMD: −0.69 [95% CI, −1.12 to −0.26], *p* = 0.002; I^2^ = 88%, *p* < 0.0001). However, no significant changes in ALT levels were found for exercise interventions of <12 weeks duration.

Subgroup analyses for BMI status revealed that the exercise intervention produced a significantly large effect in reducing ALT levels in patients with BMIs ≥30 kg/m^2^ (SMD: −0.93 [95% CI, −1.53 to −0.32], *p* = 0.003; I^2^ = 91%, *p* < 0.0001). A small effect (non-significant) on ALT levels was found following exercise in patients with a BMI of 25.0–29.9 kg/m^2^ (SMD: −0.13 [95% CI, −0.61 to 0.35], *p* = 0.60; I^2^ = 68%, *p* < 0.001).

#### 3.4.2. AST Levels

**Figure 3:**
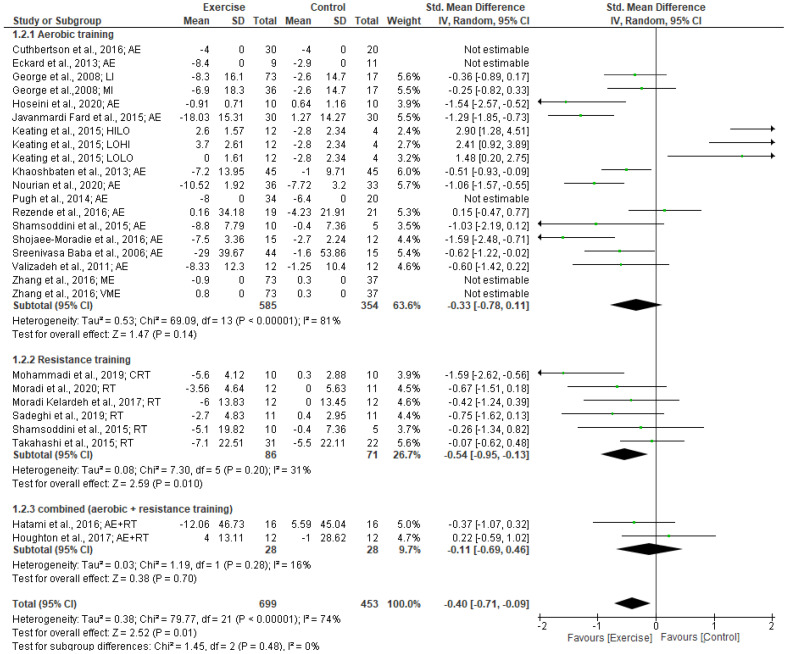
Forest plot showing study precision against the mean difference effect estimate with 95% confidence interval for AST [28,29,30,31,32,33,34,35,36,37,38,39,40,41,42,44,45,46,47,48,49,51].

Our meta-analysis of 22 studies revealed a significantly small effect of exercise training in reducing AST levels (SMD: −0.40 [95% CI, −0.71 to −0.09], *p* = 0.001; I^2^ = 74%, *p* < 0.001). The subgroup analysis by type of exercise revealed that resistance training had a significantly moderate effect on AST levels (SMD: −0.54 [95% CI, −0.95 to −0.13], *p* = 0.001; I^2^ = 31%, *p* < 0.20). There were no significant effects for either aerobic training or combined exercise training (aerobic + resistance) on AST levels (see Forest plot in Figure 3).

Subgroup analysis based on intervention duration indicated a significantly moderate effect on AST levels for intervention of ≥12 weeks (SMD: −0.59 [95% CI, −0.96 to −0.22], *p* < 0.002; I^2^ = 67%, *p* < 0.001). However, no significant changes in AST levels were found for exercise interventions of <12 weeks duration. Subgroup analyses for BMI status revealed that the exercise intervention produced a significantly small effect in reducing AST levels in patients with BMIs 25.0–29.9 kg/m^2^ (SMD: −0.27 [95% CI, −0.53 to −0.02], *p* = 0.04; I^2^ = 27%, *p* < 0.21). However, there were no significant effects on AST levels following exercise in patients with BMIs ≥30 kg/m^2^ (SMD: −0.14 [95% CI, −0.77 to 0.50], *p* = 0.67; I^2^ = 85%, *p* < 0.001).

### 3.5. Effect of Physical Activity on Glucose Metabolism Parameters

#### 3.5.1. FBG Levels

**Figure 4:**
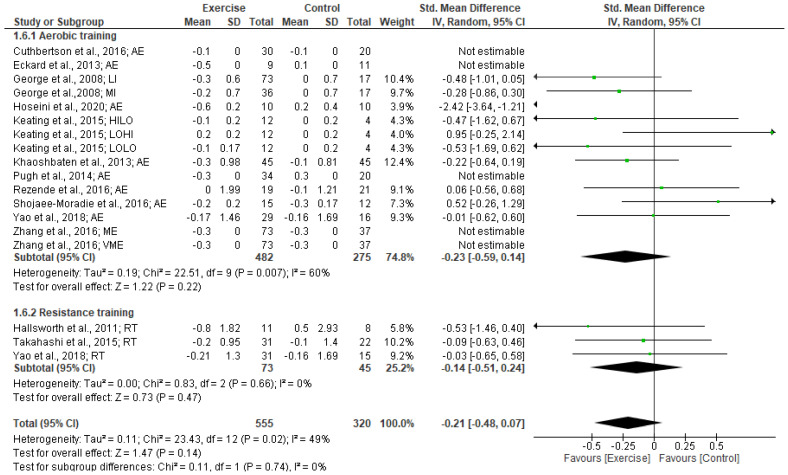
Forest plot showing study precision against the mean difference effect estimate with 95% confidence interval for FBG [30,36,37,38,40,41,42,43,46,47,49,51,52].

Our meta-analysis of 13 studies revealed no significantly small effect of exercise training on FBG levels (SMD: −0.21 [95% CI, −0.48 to 0.07], *p* = 0.14; I^2^ = 49%, *p* < 0.02). From the subgroup analysis of exercise type, no significant effects on FBG levels were found following aerobic training (SMD: −0.23 [95% CI, −0.59 to 0.14], *p* = 0.22) and resistance training (SMD: −0.14 [95% CI, −0.51 to 0.24], *p* = 0.47) (see Forest plot in Figure 4).

Subgroup analysis based on intervention duration indicated a significantly moderate effect on FBG levels for intervention of <12 weeks (SMD: −0.53 [95% CI, −0.99 to −0.07], *p* = 0.02; I^2^ = 57%, *p* < 0.02). However, no significant changes in FBG levels were found for exercise interventions of ≥12weeks duration. No significant effects of exercise on FBG levels were found from the subgroup analyses of BMI 25.0–29.9 kg/m^2^ (SMD: −0.10 [95% CI, −0.34 to 0.13], *p* = 0.38) and ≥30 kg/m^2^ (SMD: −0.34 [95% CI, −0.76 to 0.07], *p* = 0.11). 

#### 3.5.2. Insulin levels

**Figure 5:**
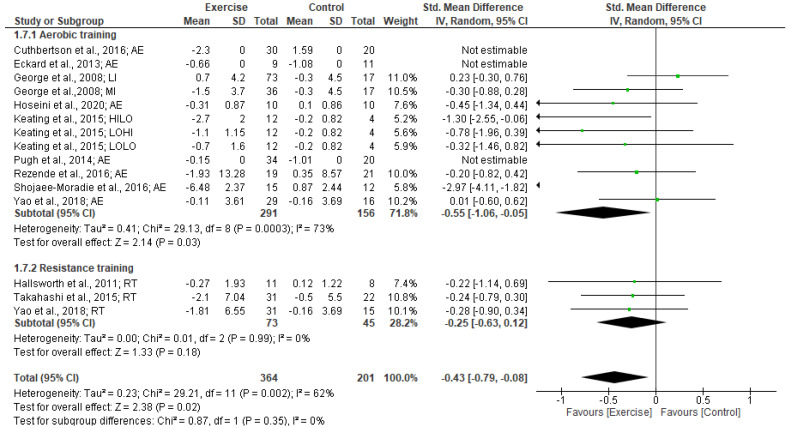
Forest plot showing study precision against the mean difference effect estimate with 95% confidence interval for insulin [30,36,37,38,40,42,43,46,49,51,52].

Our meta-analysis of 11 studies revealed a significantly small effect of exercise training in reducing insulin levels (SMD: −0.43 [95% CI, −0.79 to −0.08], *p* = 0.02; I^2^ = 62%, *p* < 0.002). The subgroup analysis by exercise type revealed that aerobic training had a significantly moderate effect in decreasing insulin (SMD: −0.55 [95% CI, −1.06 to −0.05], *p* = 0.03; I^2^ = 73%, *p* < 0.003). However, resistance training did not have a significantly small effect on insulin (SMD: −0.25 [95% CI, −0.63 to 0.12], *p* = 0.18; I^2^ = 0%, *p* < 0.99) (see Forest plot in Figure 5).

Subgroup analysis based on intervention duration indicated no significant changes in insulin were found for exercise interventions for ≥12 weeks (SMD: −0.79 [95% CI, −1.68 to 0.1], *p* = 0.08; I^2^ = 85%, *p* < 0.002) and <12 weeks duration (SMD: −0.30 [95% CI, −0.70 to 0.1], *p* = 0.14; I^2^ = 28%, *p* < 0.22).

Subgroup analyses for BMI status revealed that the exercise intervention produced a significantly moderate effect on reducing insulin in patients with BMIs ≥ 30 kg/m^2^ (SMD: −0.60 [95% CI, −1.12 to 0.08], *p* = 0.02; I^2^ = 71%, *p* < 0.005). No significant effect of exercise on FBG levels were found from the subgroup analyses of BMI 25.0–29.9 kg/m^2^ (SMD: −0.18 [95% CI, −0.46 to 0.1], *p* = 0.21; I^2^ = 0%, *p* < 0.91). 

#### 3.5.3. HOMA-IR 

**Figure 6:**
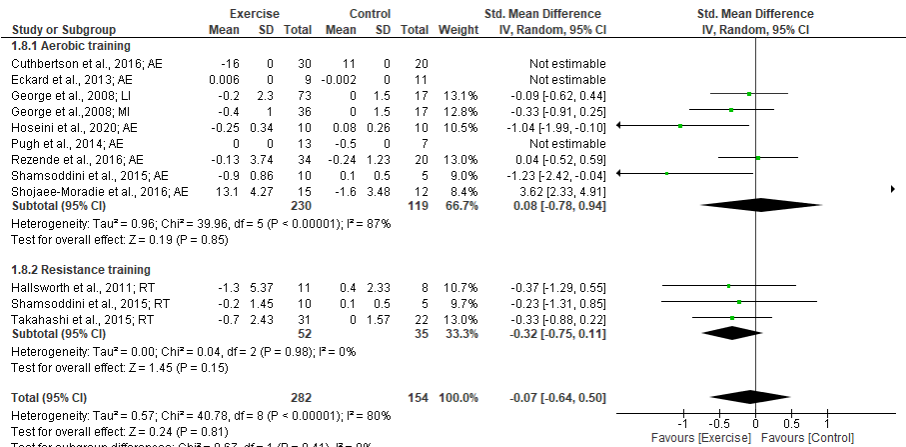
Forest plot showing study precision against the mean difference effect estimate with 95% confidence interval for HOMA-IR [30,31,36,37,38,40,42,43,49,51].

Our meta-analysis of 10 studies revealed exercise training did not change HOMA-IR levels (SMD: −0.07 [95% CI, −0.64 to 0.05], *p* = 0.81; I^2^ = 80%, *p* < 0.001).The subgroup analysis by exercise type revealed that resistance training had a small effect for decreasing HOMA-IR, although this was not significant (SMD: −0.32 [95% CI, −0.75 to 0.11], *p* = 0.15; I^2^ = %, *p* < 0.98). Aerobic training did not significantly reduce HOMA-IR (SMD: −0.08 [95% CI, −0.78 to 0.94], *p* = 0.85; I^2^ = 87%, *p* < 0.001) (see Forest plot in Figure 6).

Subgroup analysis based on intervention duration indicated a significantly small effect on HOMA-IR for <12 weeks (SMD: −0.46 [95% CI, −0.74 to −0.18], *p* = 0.001; I^2^ = 0%, *p* < 0.48). However, no significant changes in HOMA-IR were found for exercise interventions of ≥12 weeks duration (SMD: 0.97 [95% CI, −0.63 to 2.57], *p* = 0.24; I^2^ = 93%, *p* < 0.0001). Subgroup analyses for BMI status revealed that the exercise intervention produced a significantly small effect in reducing HOMA-IR in patients with BMIs 25.0–29.9 kg/m^2^ (SMD: −0.44 [95% CI, −0.78 to −0.1], *p* = 0.01; I^2^ = 0%, *p* < 0.57). No significant effect of exercise on HOMA-IR was found for patients with BMIs ≥30 kg/m^2^ (SMD: −0.04 [95% CI, −0.66 to 0.59], *p* = 0.90; I^2^ = 83%, *p* < 0.0001). 

### 3.6. Study Quality

The overall methodological quality of the included studies was estimated to be moderate to good, with a median TESTEX score of 9 (range 8–14) out of a maximum of 15. One study scored 14, one study scored 13, one study scored 12, one study scored 11, four studies scored 10, ten studies scored 9, six studies scored 8, one study scored 7, and one study scored 6 (Supplementary Table S2). Of the TESTEX criteria, the following was done particularly poorly: randomization specified (13/26 studies); allocation concealment (8/26 studies); blinding of assessor (11/26 studies); intention to treat analyses (1/26 study); and physical activity monitoring in the control groups (7/26 studies). The other criteria were each met by at least 50% of included studies.

### 3.7. Heterogeneity and Publication Bias

**Figure 7:**
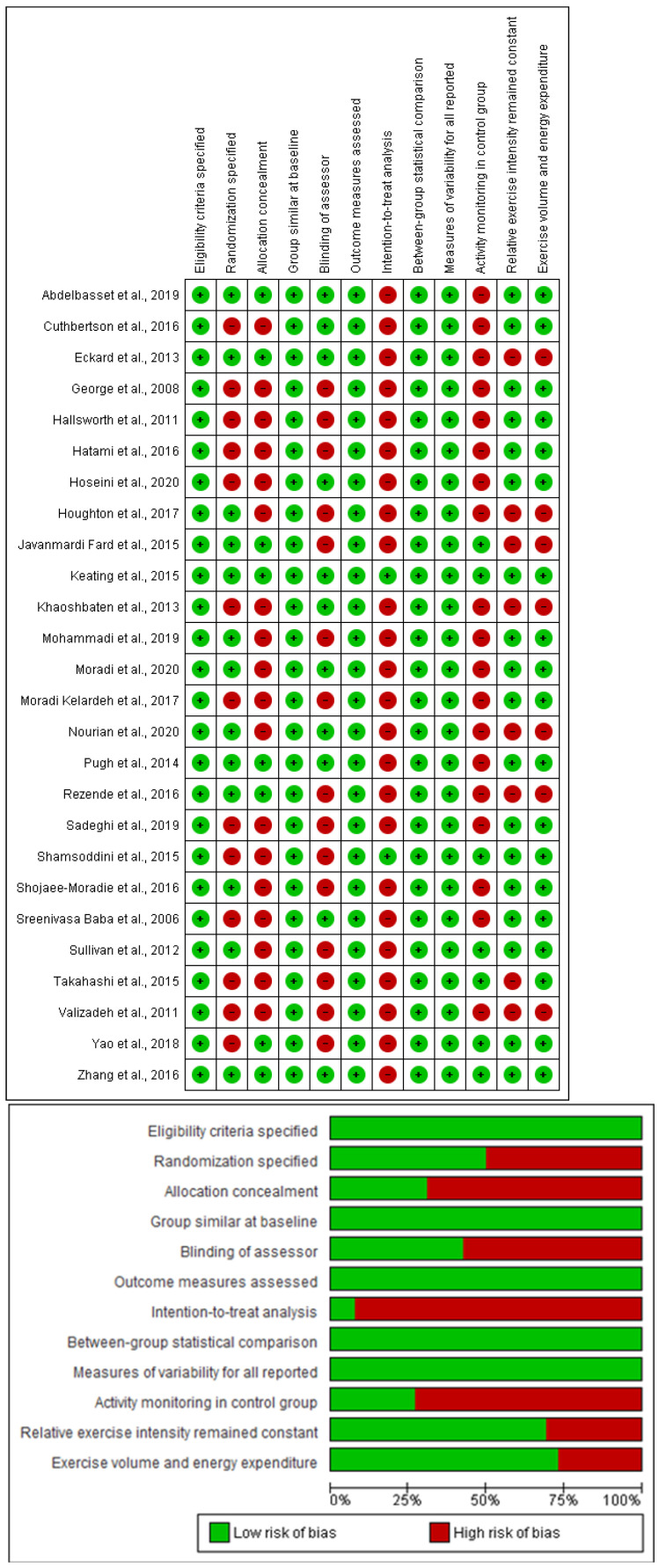
Risk of bias assessment using Cochrane risk of bias tool. Top panel—Risk of bias summary showing review author’s judgment about each risk of bias item for each included study. Bottom panel—Risk of bias graph showing review author’s judgment about each risk of bias item presented as percentages across all included studies [26,28,29,30,31,32,33,34,35,36,37,38,39,40,41,42,43,44,45,46,47,48,49,50,51,52].

Our analyses based on the effect of exercise demonstrated high heterogeneity in ALT levels (I^2^ = 88%, *p* = 0.0001) and HOMA-IR (I^2^ = 80%, *p* = 0.0001) and moderate heterogeneity in AST levels (I^2^ = 74%, *p* = 0.0001) and insulin levels (I^2^ = 62%, *p* = 0.002) However, FBG levels (I^2^ = 49%, *p* = 0.02) revealed low heterogeneity. Egger plots exhibited little to high evidence of publication bias as the standard error/mean difference plots were tightly grouped together (Supplementary Figures S1–S5). Furthermore, to show the quality of included studies, Figure 7 illustrates the results from the risk of bias assessment using the Cochrane risk of bias tool.

## 4. Discussion

The purpose of the current systematic review and meta-analysis was to investigate the effects of exercise on liver function and insulin resistance markers in patients with non-alcoholic fatty liver disease. Overall, the pooled results from the 26 RCTs (with 1316 participants) indicated that exercise training significantly reduced liver enzymes (i.e., ALT and AST) and insulin in patients with non-alcoholic fatty liver disease.

Different exercise modalities were found to be effective in reducing liver enzymes (i.e., ALT and AST) and insulin. Aerobic training and resistance training were all effective in reducing ALT, while only resistance exercise training also led to reductions in AST levels. As for insulin resistance, this was improved following aerobic training but not after resistance training. There was no specific exercise training modality that effectively reduced FBG levels in the NAFLD patients. The exercise intervention duration appeared to influence its effectiveness with <12 weeks interventions favoring reductions in FBG and HOMA-IR, while ≥12 weeks interventions favored reduction in ALT and AST levels. Additionally, BMI status impacted the effectiveness of exercise with greater reductions in AST and HOMA-IR in overweight participants, whereas obese participants had a greater reduction in ALT and insulin levels. Overall, the quality of the literature included in the meta-analyses was moderate to good. Low to moderate heterogeneity and publication bias was found within most of the analyses.

The findings from the present review are consistent with the results of the meta-analysis conducted by Li et al. [54]. This previous meta-analysis in NAFLD patients showed that exercise was effective in reducing ALT and FBG. Another meta-analysis by Wang et al. in 951 patients with NAFLD showed that aerobic training as well as resistance training led to significant reductions in ALT and AST [55]. Smart et al. conducted a meta-analysis analyzing the effects of various exercise interventions (i.e., aerobic training, resistance training, and combined exercise training) on liver enzymes in 1530 patients with NAFLD. However, in contrast to the findings from the present review, their results showed no significant changes in the levels of ALT and AST following the exercise interventions [17]. One of the possible reasons for the discrepancy between the results of the present study and the findings of Smart et al. could be the diversity of RCTs included [17]. As an example, the characteristics of the RCTs included the participant characteristics (age, BMI, and sex) and study design. While the present review included 26 studies, the review by Smart et al. included 21 studies, which further supports the possibility of the diversity of RCTs, explaining the discrepancy between results [17].

Since there was evidence that the body weight of participants influenced the effectiveness of exercise on clinical markers of NAFLD, there may be altered responses for patients based on the amount of body fat. Alternatively, the ability to perform exercise may be influenced by the amount of excess body weight, which could also impact metabolic responses and adaptations. While further research is required to explore whether the degree of “fatness” impacts the effectiveness of exercise on clinical markers of NAFLD, it has been recognized that weight loss is associated with important pathophysiological changes, specifically increased insulin sensitivity, decreased fatty acids in the liver, decreased inflammatory mechanisms, and improved levels of ALT and AST enzymes [56]. In this regard, Katsagoni et al. reported that the effects of exercise on AST and ALT depend on weight loss. It has been shown that moderate intensity aerobic exercise with higher volume (above 180 min per week) is more effective in reducing intrahepatic triglyceride than moderate intensity aerobic exercise with low volume (volume 120 to 180 min per week) [57]. The mechanism attributed to the significant decreases in ALT and AST enzymes following exercise includes increased sensitivity of insulin, increased liver oxidation, decreased activity and inhibition of lipogenic enzymes, and thus reduced liver fat [58]. As for improvement of liver lipid composition, this may be mediated by adiponectin, lipid oxidation, and increased insulin sensitivity. Hallsworth et al. reported that insulin sensitivity, circulating lipids, and energy balance may be affected by mechanistic changes in liver fat after exercise training [43].

The results from the present meta-analysis are consistent with the findings of a meta-analysis conducted by Mohammad Rahimi et al. involving 2255 NAFLD patients. The results of this previous meta-analysis showed that aerobic exercise combined with diet led to a significant improvement in not only liver enzymes but also insulin sensitivity [59]. While diet-induced weight loss as well as exercise alone is associated with improvements in insulin sensitivity and glucose tolerance, a combination of diet and exercise is expected to promote the best results [60]. Calorie restriction and intensified physical activity (PA) are usually the basic components of intensive lifestyle (ILS) interventions causing weight loss. These interventions can hinder the inception of T2D in endangered populations and reduce the risk of cardiovascular disease (CVD) in patients with T2D. Physical activity is consistently reported to be as effective as weight loss, if not more so, for T2D prevention [61].

The review findings of both short and longer duration exercise interventions improving clinical markers in patients with NAFLD were not surprising. While longer duration exercise interventions would offer the advantage of potentially greater weight loss which would positively affect these markers (mentioned above), there is strong evidence for the effectiveness of exercise interventions of a shorter duration. According to some meta-analyses and systematic reviews, patients with T2D and fewer daily hyperglycemic excursions and 0.5%–0.7% reductions in hemoglobinA1C (A1C) show improved glycemia as a results of regular aerobic exercise training [62,63]. Furthermore, short-term aerobic exercise training leads to improvement in insulin sensitivity and mitochondrial function in patients with T2D [64]. In patients with obesity and T2D, short-term aerobic exercise increased peripheral insulin sensitivity more than hepatic insulin sensitivity and thus enhanced whole-body insulin action [65]. Vigorous aerobic exercise training for 7 days increased insulin-stimulated glucose disposal and suppression of hepatic glucose production, leading to improvement of glycemia without lowering body weight [66].

The present study has some limitations that should be considered when interpreting the results. One limitation was that our selection criteria was not sensitive to the geographical region where RCTs were conducted. Furthermore, many of the studies had small sample sizes (i.e., <20 participants per group), and only studies published in English were included. However, even with the strictest selection criteria for study inclusion, it is challenging to avoid some heterogeneity [67]. Factors contributing to the heterogeneity observed may include differences in study design and settings or factors more difficult to control, such as differences in response to exercise (i.e., responders versus non-responders) [68,69]. In addition, the exercise training protocols added to the present meta-analysis included aerobic and resistance exercises that were performed with varying intensity and duration. Therefore, variation in exercise components including duration and type of exercise may have played an important role in the adaptation of liver enzymes and insulin resistance indices to exercise. Finally, only one study met the inclusion criteria that used interval training as an exercise intervention [26]. We decided not to include this study in any analysis to ensure that conclusive results from the present review could be provided. However, it should be noted that there is evidence that high-intensity interval training (HIIT) is an efficacious exercise type for improvements in liver fat [70]. Furthermore, HIIT may elicit similar improvements in liver fat compared to continuous aerobic exercise, although requiring less energy and time commitment on the part of the patient.

## 5. Conclusions

The findings of this meta-analysis support the effectiveness of exercise in improving liver function markers but not blood glucose control in NAFLD patients. Aerobic training and resistance training appear to be of value in improving the health of patients with NAFLD. Intervention duration and BMI status may influence the effect of exercise on clinical markers of NAFLD. However, further studies are required to explore this topic as well as to determine the optimal exercise prescription to maximize health in patients with NAFLD.

## Figures and Tables

**Table 2:** Exercise intervention details.

			Intervention			
Study	Groups	Mode	Frequency (Days)	Intervention Duration (Weeks)	Duration of Each Session (Min)	Intensity
Abdelbasset et al., 2019 [26]	HIIT CON	Exercise program was performed on a cycle ergometer	3/7	8	40 min	HIIT group: 80–85% VO_2_max CON group: No intervention
Cuthbertson et al., 2016 [42]	AE CON	Aerobic exercise (treadmill, cross-trainer, bike ergometer, rower)	3–5/7	12	30–45 min	AE group: 30–60% HRmax CON group: No intervention
Eckard et al., 2013 [37]	AE CON	Participants followed an 18-step program, including warm up, exercise bicycle, walking on a treadmill, various arm and leg strength exercises, and cool down, with gradual ramp up over the 6 months	4–7/7	24	20–60 min	AE group: Moderate exercise CON group: No intervention
George et al., 2008 [36]	LI MI CON	Participants were encouraged to increase both planned and incidental moderate-intensity PA to achieve at least 150 min/week for general health and to target more than 200 min/week for weight loss	3/7 6/7	4 10	45–60 min	LI group: 35–40% VO_2_max MI group: 70–75% VO_2_max CON group: No intervention
Hallsworth et al., 2011 [43]	RT CON	The program consisted of eight exercises: biceps curl; calf raise; triceps press; chest press; seated hamstrings curl; shoulder press; leg extension; and lateral pull down	3/7	8	45–60 min	RT group: 50–70% 1RM CON group: No intervention
Hatami et al., 2016 [35]	AE + RT CON	The aerobic training section consisted of rhythmic movements and the resistance training section consisted of biceps, triceps, pectoralis, quadriceps, and hamstring muscles in the position of dumbbell biceps (seated concentration curl), dumbbell triceps (triceps kickback), bench press, knee extension, and knee flexion	3/7	8	40 min	AE + RT group: 3 sets/8–12 reps at 60–80% 1RM 65–85% HRmax CON group: No intervention
Hoseini et al., 2020 [38]	AE CON	Walking and jogging/running	3/7	8	20–40 min	AE group: 60–75%, HRmax CON group: No intervention
Houghton et al., 2017 [44]	AE + RT CON	The exercise program consisted of aerobic (cycling) and resistance training	3/7	12	60 min	AE + RT group: Moderate vigorous Activity CON group: No intervention
Javanmardi Fard et al., 2015 [45]	AE CON	The physical activities included jogging, cycling, aerobic exercise or any other activity that is of a similar intensity	4–5/7	12	30 min	AE group: Moderate physical activities CON group: No intervention
Keating et al., 2015 [46]	HILO LOHI LOLO CON	Performed continuous cycling on the ergometer/brisk walk	3–4/7	8	45–60 min	LO:HI group: 50% VO_2_peak HI:LO group: 70% VO_2_peak LO:LO group: 50% VO_2_peak CON group: No intervention
Khaoshbaten et al., 2013 [47]	AE CON	Walking/running	3/7	12	30 min	AE group: Maximal heart rate CON group: No intervention
Mohammadi et al., 2019 [34]	CRT CON	The resistance training included three circles with nine stations per circle	3/7	12	30–45 min	CRT group: 3 sets/10–20 reps at 40–80% 1RM CON group: No intervention
Moradi Kelardeh et al., 2017 [33]	RT CON	Knee extension, bench press, incline bench press, seated row, dead lift, pulley crunches, lat pull-downs, calf raise, hamstring curl, press behind neck, upright row, arm curl	3/7	12	45–60 min	RT group: 1–3 sets/2–20 reps at 40–95% 1RM CON group: No intervention
Moradi Kelardeh et al., 2020 [39]	RT CON	Knee extension, bench press, incline bench press, seated row, dead lift, pulley crunches, lat pull-downs, calf raise, hamstring curl, press behind neck, upright row, arm curl	3/7	12	60–70 min	RT group: 1–4 sets/2–20 reps at 40–95% 1RM CON group: No intervention
Nourian et al., 2020 [48]	AE CON	The educational content of each session was presented to participants by using a short lecture, slide show, pamphlet, and discussion.	5/7	8	30–60 min	AE group: Lifestyle modification education training CON group: No intervention
Pugh et al., 2014 [49]	AE CON	Exercise training comprised a combination of treadmill and cycle ergometer-based exercise, which progressively increased in both intensity and duration throughout the course of the intervention	3–5/7	12	30–45 min	AE group: 30–60 HRmax CON group: No intervention
Rezende et al., 2016 [40]	AE CON	Aerobic exercise on treadmill	2/7	24	120 min	AE group: Increased from VAT up to 10% below RCP CON group: No intervention
Sadeghi et al., 2019 [32]	RT CON	These exercises include TRX plank on elbows, TRX T deltoid fly, TRX chest press, TRX high row, TRX triceps press, TRX biceps curl, TRX squat, and TRX hip press	3/7	8	60 min	RT group: 3 sets/8 reps at 70–100% 1RM CON group: No intervention
Shamsoddini et al., 2015 [31]	AE RT CON	Running on treadmill/seven exercises with machine: triceps press, biceps curl, calf raise, leg press, leg extension, lat pull down, and sit-ups	3/7	8	45 min	AE group: 60–75% HRmax RT group: 50–70% 1RM CON group: No intervention
Shojaee-Moradie et al., 2016 [30]	AE CON	Types of activities were either gym-based aerobic plus resistance exercise or outdoor aerobic activities and resistance exercise	4–5/7	16	20–60 min	AE group: 40–60% HRmax CON group: No intervention
Sreenivasa Baba et al., 2006 [29]	AE CON	Brisk walking/jogging or rhythmic aerobic exercises set to beat music	5/7	12	20–45 min	AE group: 60–70% HRmax CON group: No intervention
Sullivan et al., 2012 [50]	AE CON	Brisk walk/walking on a treadmill	5/7	16	30–60 min	AE group: 45–55% VO_2_peak CON group: No intervention
Takahashi et al., 2015 [51]	RT CON	Resistance exercise comprising push-ups and squats at the beginning of the study.	3/7	12	20–30 min	RT group: 3 sets/10 reps CON group: No intervention
Valizadeh et al., 2011 [28]	AE CON	Walking/running	5/7	8	45 min	AE group: 50–70% VO_2_max CON group: No intervention
Yao et al., 2018 [52]	AE RT CON	Walking/running Elastic band was used as the load instrument	3/7	22	40–60 min	AE group: 60–70% HRmax RT group: 3 sets/10 reps; 60–70% 1RM CON group: No intervention
Zhang et al., 2016 [41]	ME VME CON	Jogged on a treadmill and gradually increased exercise intensity	5/7	48	30 min	ME group: 65–80% HRmax CON group: No intervention

Note: The control group received no training. Abbreviations: VAT, ventilator anaerobic threshold; RCP, respiratory compensation point; CON, control; AE, aerobic; RT, resistance training; ME, moderate exercise; VME, vigorous–moderate exercise; HILO, high intensity, low volume aerobic exercise; LOHI, low to moderate intensity, high volume aerobic exercise; LOLO, low to moderate intensity, low volume aerobic exercise; LI, low intensity; MI, moderate intensity; HIIT, high-intensity interval training; HRmax, maximum heart rate; 1RM, 1 repetition maximum; VO_2_peak, peak oxygen uptake; VO_2_ma, maximal oxygen consumption.

## Data Availability

No new data were created or analyzed in this study. Data sharing is not applicable to this article.

## Supplementary Materials

The following supporting information can be downloaded at: https://www.mdpi.com/article/10.3390/jcm12083011/s1, Figure S1: Funnel plot for ALT levels; Figure S2: Funnel plot for AST levels; Figure S3: Funnel plot for FBG levels; Figure S4: Funnel plot for insulin; Figure S5: Funnel plot for HOMA-IR; Table S1: PRISMA Checklist; Table S2. Study quality assessment; Table S3: Search strategy terms.
