# Supplementary material for: Effect of Exercise on Liver Function and Insulin Resistance Markers in Patients with Non-Alcoholic Fatty Liver Disease: A Systematic Review and Meta-Analysis of Randomized Controlled Trials
Source: J Clin Med. 2023 Apr 20;12(8):3011. doi: 10.3390/jcm12083011 (PMC10143548; doi:10.3390/jcm12083011)
Supplement: Supplementary file 1 [file jcm-12-03011-s001.zip › jcm-2318287-supplementary.pdf]

Supplementary Materials

Table S1: Prisma Statement.

Section/topic	#	Checklist item	Reported on page #
**TITLE**			
Title	1	Identify the report as a systematic review, meta-analysis, or both.	1
**ABSTRACT**			
Structured summary	2	Provide a structured summary including, as applicable: background; objectives; data sources; study eligibility criteria, participants, and interventions; study appraisal and synthesis methods; results; limitations; conclusions and implications of key findings; systematic review registration number.	2
**INTRODUCTION**			
Rationale	3	Describe the rationale for the review in the context of what is already known.	4,5
Objectives	4	Provide an explicit statement of questions being addressed with reference to participants, interventions, comparisons, outcomes, and study design (PICOS).	5
**METHODS**			
Protocol and registration	5	Indicate if a review protocol exists, if and where it can be accessed (e.g., Web address), and, if available, provide registration information including registration number.	n/a
Eligibility criteria	6	Specify study characteristics (e.g., PICOS, length of follow-up) and report characteristics (e.g., years considered, language, publication status) used as criteria for eligibility, giving rationale.	5,6
Information sources	7	Describe all information sources (e.g., databases with dates of coverage, contact with study authors to identify additional studies) in the search and date last searched.	5
Search	8	Present full electronic search strategy for at least one database, including any limits used, such that it could be repeated.	Supplement File
Study selection	9	State the process for selecting studies (i.e., screening, eligibility, included in systematic review, and, if applicable, included in the meta-analysis).	5,6
Data collection process	10	Describe method of data extraction from reports (e.g., piloted forms, independently, in duplicate) and any processes for obtaining and confirming data from investigators.	6
Data items	11	List and define all variables for which data were sought (e.g., PICOS, funding sources) and any assumptions and simplifications made.	6
Risk of bias in individual studies	12	Describe methods used for assessing risk of bias of individual studies (including specification of whether this was done at the study or outcome level), and how this information is to be used in any data synthesis.	n/a
Summary measures	13	State the principal summary measures (e.g., risk ratio, difference in means).	7,8
Synthesis of results	14	Describe the methods of handling data and combining results of studies, if done, including measures of consistency (e.g., I^2) for each meta-analysis.	7,8
Risk of bias across studies	15	Specify any assessment of risk of bias that may affect the cumulative evidence (e.g., publication bias, selective reporting within studies).	7,8
Additional analyses	16	Describe methods of additional analyses (e.g., sensitivity or subgroup analyses, meta-regression), if done, indicating which were pre-specified.	7,8
**RESULTS**			
Study selection	17	Give numbers of studies screened, assessed for eligibility, and included in the review, with reasons for exclusions at each stage, ideally with a flow diagram.	9 & Fig 1
Study characteristics	18	For each study, present characteristics for which data were extracted (e.g., study size, PICOS, follow-up period) and provide the citations.	Table 1
Risk of bias within studies	19	Present data on risk of bias of each study and, if available, any outcome level assessment (see item 12).	n/a
Results of individual studies	20	For all outcomes considered (benefits or harms), present, for each study: (a) simple summary data for each intervention group (b) effect estimates and confidence intervals, ideally with a forest plot.	Figs 2-6<br>Table 2
Synthesis of results	21	Present results of each meta-analysis done, including confidence intervals and measures of consistency.	Figs 2-6<br>Table 2
Risk of bias across studies	22	Present results of any assessment of risk of bias across studies (see Item 15).	Supp Fig 1-5
Additional analysis	23	Give results of additional analyses, if done (e.g., sensitivity or subgroup analyses, meta-regression [see Item 16]).	Table 2

**DISCUSSION**			
Summary of evidence	24	Summarize the main findings including the strength of evidence for each main outcome; consider their relevance to key groups (e.g., healthcare providers, users, and policy makers).	15-19
Limitations	25	Discuss limitations at study and outcome level (e.g., risk of bias), and at review-level (e.g., incomplete retrieval of identified research, reporting bias).	19
Conclusions	26	Provide a general interpretation of the results in the context of other evidence, and implications for future research.	20
**FUNDING**			
Funding	27	Describe sources of funding for the systematic review and other support (e.g., supply of data); role of funders for the systematic review.	20

Table S2. Study quality assessment of included studies by the tool for the assessment of study quality in exercise (TESTEX).

**Study<br>Random**	**Eligibility criteria specified**	**Randomization specified**	**Allocation concealment**	**Group similar at baseline**	**Blinding of assessor**	**Outcome measures assessed in 85% of patients #**	**Intention-to-treat analysis**	**Between-group statistical comparisons were reported ***	**Point measures and measures of variability for all reported outcome measures**	**Activity monitoring in control group**	**Relative exercise intensity remained constant**	**Exercise volume and energy expenditure**	**Overall TESTEX (/15)**
Abdelbasset, 2019	1	1	1	1	1	2	0	2	1	0	1	1	12
Cuthbertson, 2016	1	0	0	1	1	1	0	2	1	0	1	1	9
Eckard, 2013	1	1	1	1	1	1	0	2	1	0	0	0	9
George, 2008	1	0	0	1	0	2	0	2	1	0	1	1	9
Hallsworth, 2011	1	0	0	1	0	2	0	2	1	0	1	1	9
Hatami, 2016	1	0	0	1	0	1	0	2	1	0	1	1	8
Hoseini, 2020	1	0	0	1	1	1	0	2	1	0	1	1	9
Houghton, 2017	1	1	0	1	0	2	0	2	1	0	0	0	8
Javanmardi Fard, 2015	1	1	1	1	0	2	0	2	1	1	0	0	10
Keating, 2015	1	1	1	1	1	2	1	2	1	1	1	1	14
Khaoshbaten, 2013	1	0	0	1	1	1	0	2	1	0	0	0	7
Mohammadi, 2019	1	1	0	1	0	1	0	2	1	0	1	1	9
Moradi Kelardeh, 2017	1	0	0	1	0	1	0	2	1	0	1	1	8
Moradi, 2020	1	1	0	1	1	1	0	2	1	0	1	1	10
Nourian, 2020	1	1	0	1	1	1	0	2	1	0	0	0	8
Pugh, 2014	1	1	1	1	1	1	0	2	1	0	1	1	11
Rezende, 2016	1	1	1	1	0	2	0	2	1	0	0	0	9
Sadeghi, 2019	1	0	0	1	0	1	0	2	1	0	1	1	8
Shamsoddini, 2015	1	0	0	1	0	1	0	2	1	1	1	1	9
Shojaee-Moradie, 2016	1	1	0	1	0	1	0	2	1	0	1	1	9
Sreenivasa Baba, 2006	1	0	0	1	1	1	0	2	1	0	1	1	9
Sullivan, 2012	1	1	0	1	0	1	0	2	1	1	1	1	10
Takahashi, 2015	1	0	0	1	0	1	0	2	1	1	0	1	8
Valizadeh, 2011	1	0	0	1	0	1	0	2	1	0	0	0	6
Yao, 2018	1	0	1	1	0	1	0	2	1	1	1	1	10
Zhang, 2016	1	1	1	1	1	2	0	2	1	1	1	1	13

Total out of 15 points. # Three points possible—1 point if adherence >85%, 1 point if adverse events reported, 1 point if exercise attendance is reported. * Two points possible—1 point if primary outcome is reported, 1 point if all other outcome reported.

Egger Plots

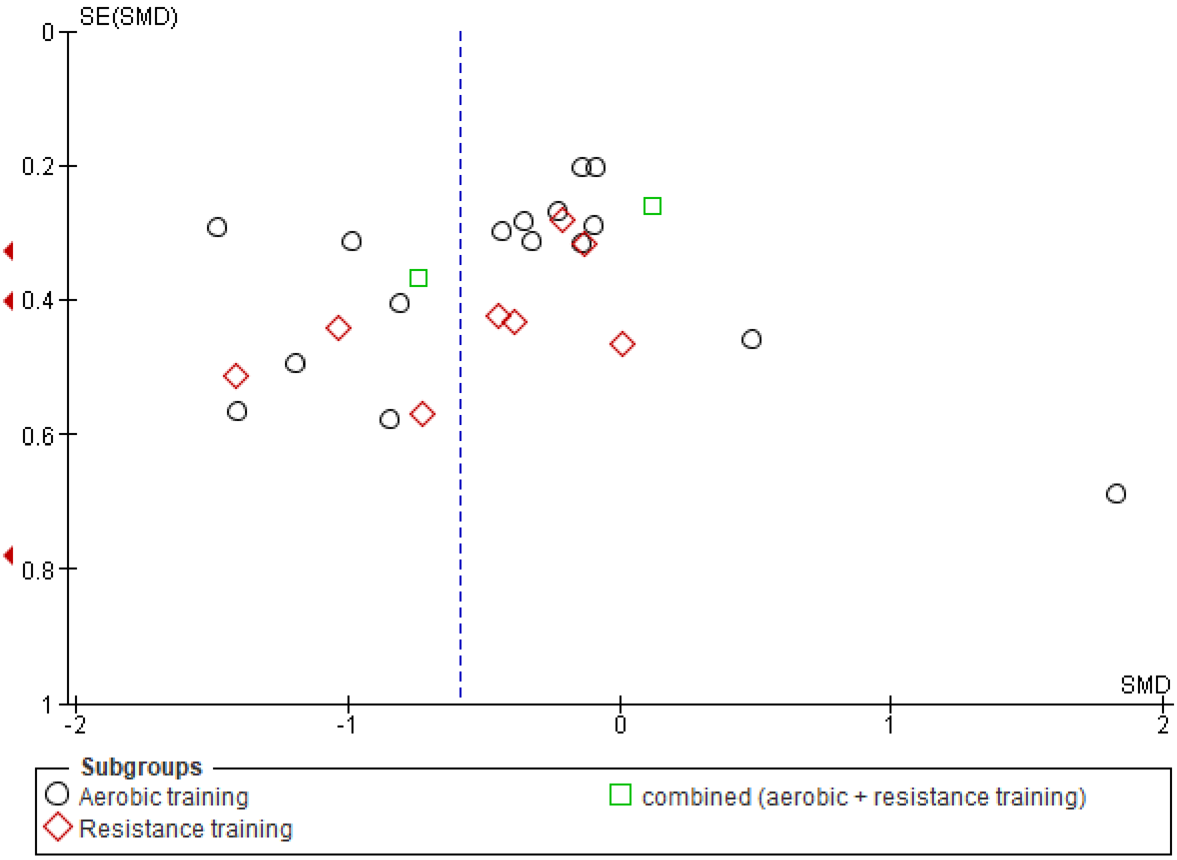

Figure S1. Funnel plot showing study precision against the mean difference effect estimate with 95% confidence interval for ALT. SE, standard error; MD, mean difference.

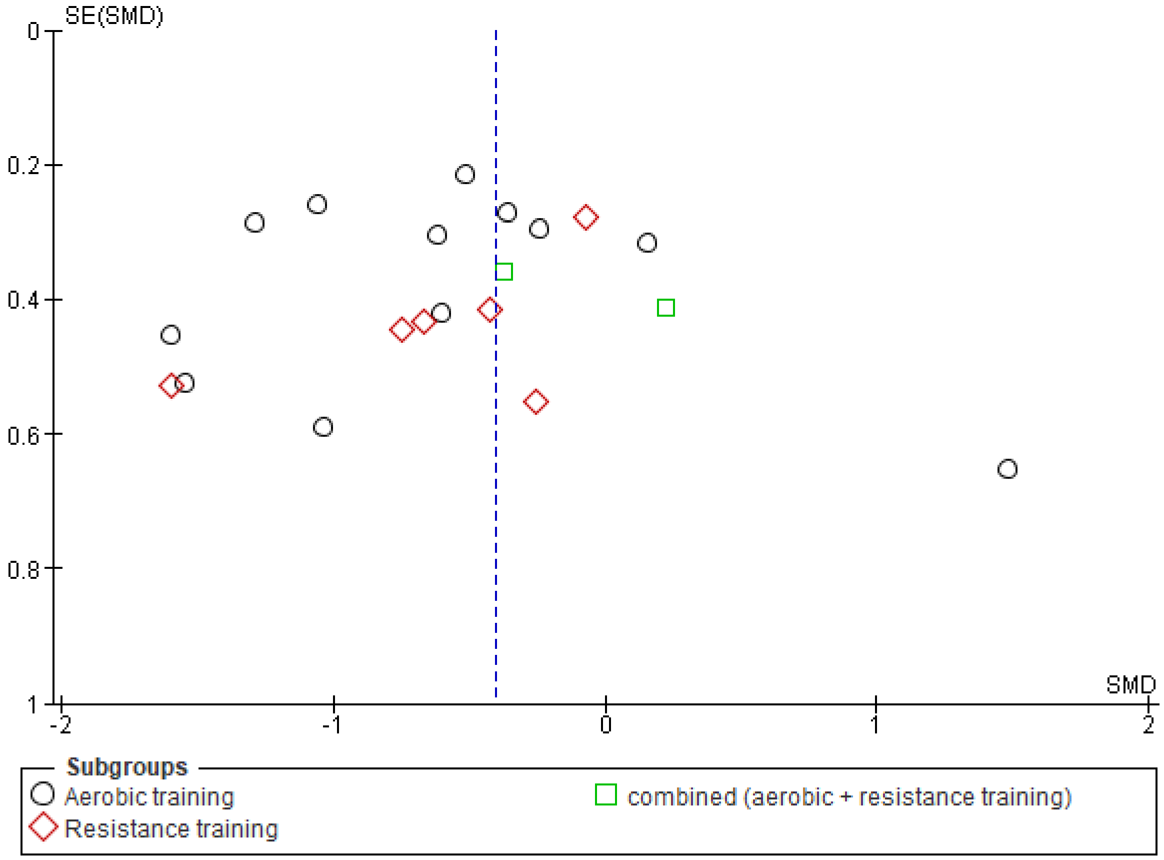

Figure S2. Funnel plot showing study precision against the mean difference effect estimate with 95% confidence interval for AST. SE, standard error; MD, mean difference.

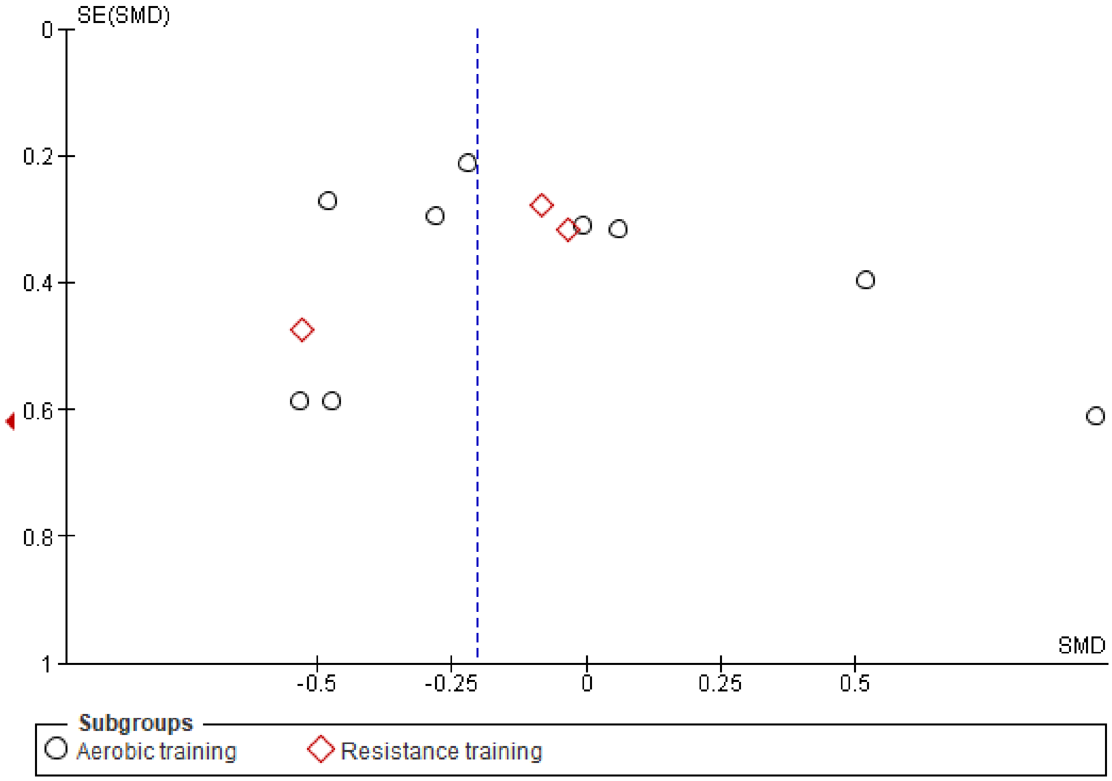

Figure S3. Funnel plot showing study precision against the mean difference effect estimate with 95% confidence interval for FBG. SE, standard error; MD, mean difference.

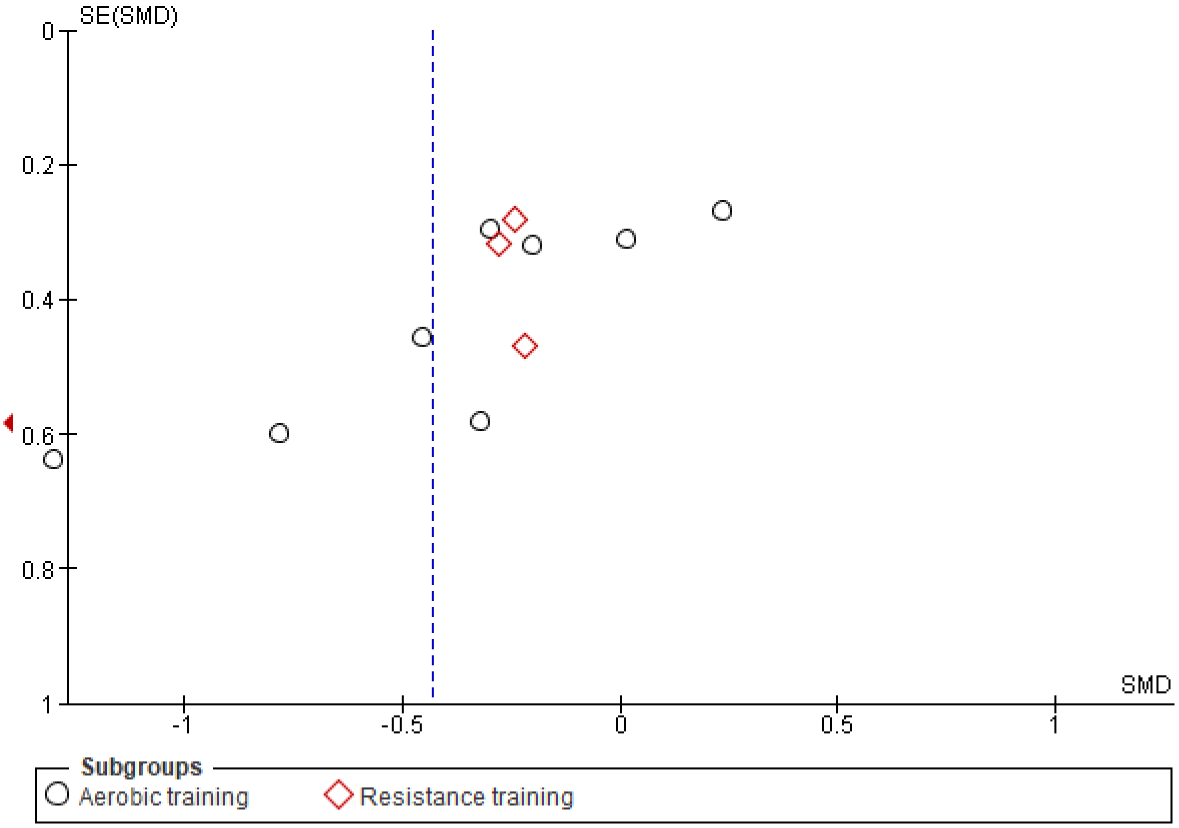

Figure S4. Funnel plot showing study precision against the mean difference effect estimate with 95% confidence interval for Insulin. SE, standard error; MD, mean difference.

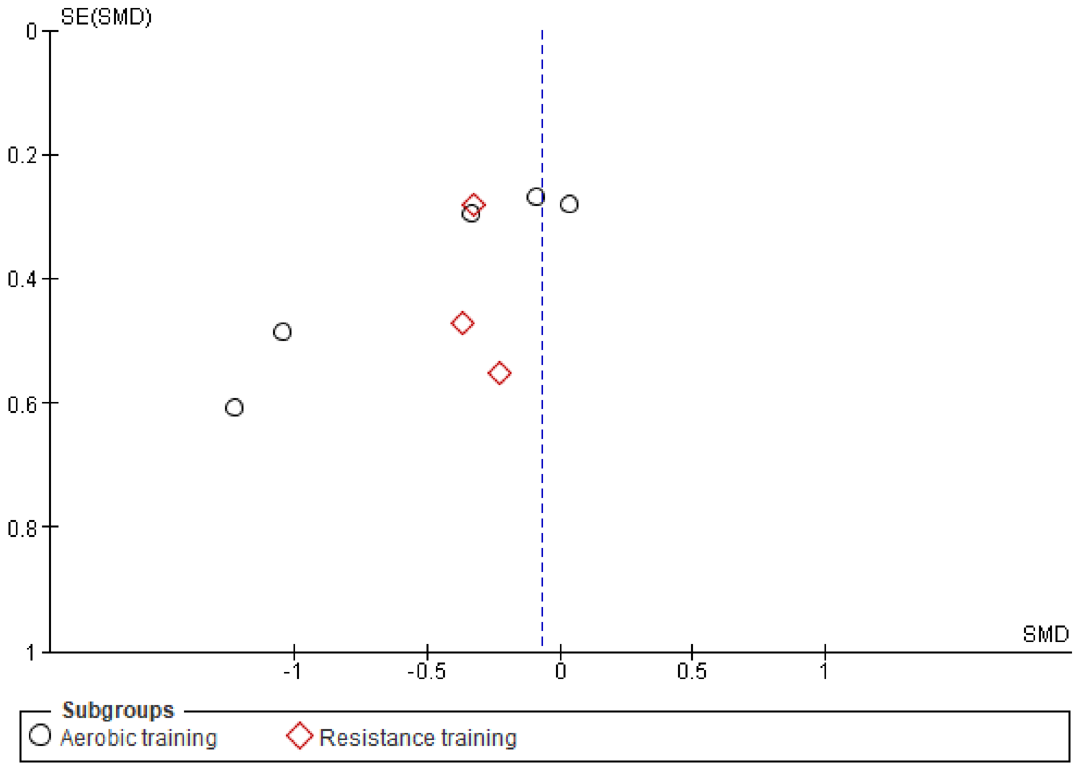

Figure S5. Funnel plot showing study precision against the mean difference effect estimate with 95% confidence interval for HOMA-IR. SE, standard error; MD, mean difference.

Table S3. Supplementary Search Strategy Terms.

Groups	Descriptors
Outcome	Liver Steatosis OR Non-Alcoholic Fatty Liver Disease OR NAFLD OR Fatty Liver OR Liver OR Hepatic OR NASH OR Aminotransferase OR Liver Enzymes OR AST OR ALT OR FBG OR Fating Insulin OR HOMA-IR OR HbA1c
Intervention	Exercise OR physical activity OR Training, Exercise OR sport OR aerobic training OR anaerobic training OR endurance training OR resistance training OR interval training OR walking OR muscle strengthening OR strength training OR combined training OR walking OR circuit training OR circuit OR weight training OR chronic training OR lifestyle activity OR home training OR water exercise OR tai chi training
Setting	Randomized controlled trial OR controlled clinical trial OR randomized controlled trials OR random allocation OR double blind method OR single blind method OR clinical trial OR clinical trials OR random

WEB OF SCIENCE

	Descriptors
**#1**	TS=("Exercise ") OR TS=("Physical Activity") OR TS=("Training, Exercise") OR TS=("Sport") OR TS=("aerobic training") OR TS=("anaerobic training") OR TS=("endurance training") OR TS=("resistance training") OR TS=("interval training") OR TS=("walking") OR TS=("muscle strengthening") OR TS=("strength training") OR TS=("combined training") OR TS=("circuit training") OR TS=("circuit") OR TS=("weight training") OR TS=("chronic training") OR TS=("lifestyle activity") OR TS=("home training") OR TS=("water exercise") OR TS=("tai chi")
**#2**	TS=("Liver Steatosis") OR TS=(" Non-Alcoholic Fatty Liver Disease") OR TS=("NAFLD") OR TS=(" Fatty Liver") OR TS=("Hepatic") OR TS=("NASH") OR TS=("Aminotransferase") OR TS=("Liver Enzymes") OR TS=("AST") OR TS=("ALT") OR TS=("FBG") OR TS=("Fating Insulin") OR TS=(" HOMA-IR") OR TS=("HbA1c")
**#3**	TS=(Randomized controlled trial) OR TS=(controlled clinical trial) OR TS=(randomized controlled trials) OR TS=(random allocation) OR TS=(double blind method) OR TS=(single blind method) OR TS=(clinical trial) OR TS=(clinical trials) OR TS=(random)
**#4**	**#1** AND **#2** AND **#3**

EMBASE

	Descriptors
**#1**	'exercise'/exp OR exercise OR (physical AND ('activity'/exp OR activity)) OR (training, AND ('exercise'/exp OR exercise)) OR 'sport'/exp OR sport OR (aerobic AND ('training'/exp OR training)) OR (anaerobic AND ('training'/exp OR training)) OR (('endurance'/exp OR endurance) AND ('training'/exp OR training)) OR (('resistance'/exp OR resistance) AND ('training'/exp OR training)) OR (interval AND ('training'/exp OR training)) OR 'walking'/exp OR walking OR (('muscle'/exp OR muscle) AND strengthening) OR (strength AND ('training'/exp OR training)) OR (combined AND ('training'/exp OR training)) OR (circuit AND ('training'/exp OR training)) OR (weight AND ('training'/exp OR training)) OR (chronic AND ('training'/exp OR training)) OR (lifestyle AND ('activity'/exp OR 'activity')) OR (home AND ('training'/exp OR training)) OR (water AND (exercise /exp OR exercise)) OR (tai chi AND ('training'/exp OR training))

**#2**	liver AND ('steatosis'/exp OR steatosis)) AND 'non-alcoholic fatty liver disease') OR (('liver'/exp OR liver) AND ('enzymes'/exp OR enzymes)) AND ('NAFLD'/exp OR NAFLD)) AND ('hepatic'/exp OR hepatic)) OR (('fatty'/exp OR fatty) AND (' liver'/exp OR liver)) AND ('NASH'/exp OR NASH)) AND ('Aminotransferase'/exp OR Aminotransferase)) AND ('AST'/exp OR AST)) AND ('ALT'/exp OR ALT)) AND ('FBG'/exp OR FBG)) OR (('fasting'/exp OR fasting) AND ('insulin'/exp OR insulin)) AND ('HOMA-IR'/exp OR HOMA-IR))
**#3**	(randomized AND controlled AND ('trial'/exp OR trial)) OR (controlled AND ('clinical'/exp OR clinical) AND ('trial'/exp OR trial)) OR (randomized AND controlled AND trials) OR (random AND allocation) OR (double AND ('blind'/exp OR blind) AND ('method'/exp OR method)) OR (single AND ('blind'/exp OR blind) AND ('method'/exp OR method)) OR (('clinical'/exp OR clinical) AND ('trial'/exp OR trial)) OR (('clinical'/exp OR clinical) AND trials) OR (random)
**#4**	**#1** AND **#2** AND **#3**

COCHRANE

	**Descriptors**
**#1**	Me ("Exercise") or ("Physical Activity"):ti,ab,kw or ("Training, Exercise"):ti,ab,kw or ("Sport"):ti,ab,kw or ("aerobic training"):ti,ab,kw or ("anaerobic training"):ti,ab,kw or ("endurance training"):ti,ab,kw or ("resistance training"):ti,ab,kw or ("interval training"):ti,ab,kw or ("walking"):ti,ab,kw or ("muscle strengthening"):ti,ab,kw or ("strength training"):ti,ab,kw or ("combined training"):ti,ab,kw or ("circuit training"):ti,ab,kw or ("weight training"):ti,ab,kw or ("chronic training"):ti,ab,kw or ("lifestyle activity"):ti,ab,kw or ("home training"):ti,ab,kw or ("water exercise"):ti,ab,kw or ("tai chi training"):ti,ab,kw
**#2**	Me ("Liver Steatosis") or ("Non-Alcoholic Fatty Liver Disease"):ti,ab,kw or ("NAFLD "):ti,ab,kw or ("Fatty Liver"):ti,ab,kw or ("Hepatic"):ti,ab,kw or ("NASH"):ti,ab,kw or ("Aminotransferase"):ti,ab,kw or ("Liver Enzymes"):ti,ab,kw or ("AST"):ti,ab,kw or ("ALT"):ti,ab,kw or ("FBG"):ti,ab,kw or ("Fating Insulin"):ti,ab,kw or ("HOMA-IR"):ti,ab,kw or ("HbA1c"):ti,ab,kw
**#3**	**#1** AND **#2**

PUBMED

	**Descriptors**
**#1**	(((((((("liver steatosis"[MeSH Terms] OR ("liver"[All Fields] AND "steatosis"[All Fields]) OR "liver steatosis"[All Fields]) OR ("liver steatosis"[MeSH Terms] OR ("liver"[All Fields] AND "steatosis"[All Fields]) OR " liver steatosis"[All Fields] OR ("fatty liver"[MeSH Terms] OR ("fatty"[All Fields] AND "liver"[All Fields]) OR " fatty liver"[All Fields]) OR ("fatty liver"[MeSH Terms] OR ("fatty"[All Fields] AND "liver"[All Fields]) OR "fatty liver"[All Fields] OR ("liver enzymes"[MeSH Terms] OR ("liver"[All Fields] AND "enzymes"[All Fields]) OR "liver enzymes"[All Fields]) OR ("liver enzymes"[MeSH Terms] OR ("liver"[All Fields] AND "enzymes"[All Fields]) OR " liver enzymes"[All Fields] OR ("non-alcoholic"[All Fields] AND "fatty"[All Fields] AND "liver"[All Fields]) AND "disease"[All Fields]) OR " non-alcoholic fatty liver disease "[All Fields])) OR ("hepatic"[MeSH Terms] OR "hepatic"[All Fields])) OR ("aminotransferase"[MeSH Terms] OR "aminotransferase"[All Fields])) OR ("alanine transaminase"[MeSH Terms] OR "alanine transaminase"[All Fields])) OR ("aspartate transaminase"[MeSH Terms] OR "aspartate transaminase"[All Fields])) OR ("aminotransferase"[MeSH Terms] OR "aminotransferase"[All Fields])) OR ("HOMA-IR"[MeSH Terms] OR " HOMA-IR"[All Fields])) OR ("fasting"[All Fields] AND "blood"[All Fields] AND "glucose"[All Fields])) OR "fasting blood glucose"[All Fields])) OR ("fasting"[All Fields] AND "insulin"[All Fields])) OR "fasting insulin"[All Fields])) OR ("HbA1c"[MeSH Terms] OR "

	HbA1c"[All Fields]))
**#2**	((((((((("exercise"[MeSH Terms] OR "exercise"[All Fields]) OR ("exercise"[MeSH Terms] OR "exercise"[All Fields] OR ("physical"[All Fields] AND "activity"[All Fields]) OR "physical activity"[All Fields])) OR ("exercise"[MeSH Terms] OR "exercise"[All Fields] OR ("training"[All Fields] AND "activity"[All Fields]) OR " lifestyle activity "[All Fields])) OR ("exercise"[MeSH Terms] OR "exercise"[All Fields] OR ("training"[All Fields] AND "exercise"[All Fields]) OR "training, exercise"[All Fields])) OR ("sports"[MeSH Terms] OR "sports"[All Fields] OR "sport"[All Fields])) OR (aerobic[All Fields] AND ("education"[Subheading] OR "education"[All Fields] OR "training"[All Fields] OR "education"[MeSH Terms] OR "training"[All Fields]))) OR (anaerobic[All Fields] AND ("education"[Subheading] OR "education"[All Fields] OR "training"[All Fields] OR "education"[MeSH Terms] OR "training"[All Fields]))) OR (combined training [All Fields] AND("education"[Subheading] OR "education"[All Fields] OR "training"[All Fields] OR "education"[MeSH Terms] OR "training"[All Fields]))) OR (circuit training [All Fields] AND("education"[Subheading] OR "education"[All Fields] OR "training"[All Fields] OR "education"[MeSH Terms] OR "training"[All Fields]))) OR (weight training [All Fields] AND("education"[Subheading] OR "education"[All Fields] OR "training"[All Fields] OR "education"[MeSH Terms] OR "training"[All Fields]))) OR (chronic training [All Fields] AND("education"[Subheading] OR "education"[All Fields] OR "training"[All Fields] OR "education"[MeSH Terms] OR "training"[All Fields]))) OR (home training [All Fields] AND("education"[Subheading] OR "education"[All Fields] OR "training"[All Fields] OR "education"[MeSH Terms] OR "training"[All Fields]))) OR (water exercise [All Fields] AND("education"[Subheading] OR "education"[All Fields] OR "training"[All Fields] OR "education"[MeSH Terms] OR "training"[All Fields]))) OR (tai chi [All Fields] AND ("education"[Subheading] OR "education"[All Fields] OR "training"[All Fields] OR "education"[MeSH Terms] OR "training"[All Fields]))) OR (endurance[All Fields] AND ("education"[Subheading] OR "education"[All Fields] OR "training"[All Fields] OR "education"[MeSH Terms] OR "training"[All Fields]))) OR ("resistance training"[MeSH Terms] OR ("resistance"[All Fields] AND "training"[All Fields]) OR "resistance training"[All Fields])) OR (interval[All Fields] AND ("education"[Subheading] OR "education"[All Fields] OR "training"[All Fields] OR "education"[MeSH Terms] OR "training"[All Fields]))) OR ("walking"[MeSH Terms] OR "walking"[All Fields])) OR (("muscles"[MeSH Terms] OR "muscles"[All Fields] OR "muscle"[All Fields]) AND strengthening[All Fields])
**#3**	((((((((("Randomized Controlled Trial"[Publication Type] OR "Controlled Clinical Trial"[Publication Type]) OR "Randomized Controlled Trials as Topic"[Mesh]) OR "Random Allocation"[Mesh]) OR "Double-Blind Method"[Mesh]) OR "Single-Blind Method"[Mesh]) OR "Clinical Trial"[Publication Type]) OR ("clinical trial"[Publication Type] OR "clinical trials as topic"[MeSH Terms] OR "clinical trials"[All Fields])) OR ("random allocation"[MeSH Terms] OR ("random"[All Fields] AND "allocation"[All Fields]) OR "random allocation"[All Fields] OR "random"[All Fields])
**#4**	**#1** AND **#2** AND #3

SCOPUS

	**Descriptors**
#1	*((TITLE-ABS-KEY (exercise)) OR (TITLE-ABS-KEY (physical AND activity)) OR (TITLE-ABS-KEY (training, AND exercise)) OR (TITLE-ABS-KEY (sport)) OR (TITLE-ABS-KEY (aerobic AND training)) OR (TITLE-ABS-KEY (anaerobic AND training)) OR (TITLE-ABS-KEY (endurance AND training)) OR (TITLE-ABS-KEY (resistance AND training)) OR (TITLE-ABS-KEY (interval AND training))) OR (TITLE-ABS-KEY (walking)) OR (TITLE-ABS-KEY (muscle AND strengthening)) OR (TITLE-ABS-KEY (strength AND training))) OR (TITLE-ABS-KEY (combined AND training))) OR (TITLE-ABS-KEY (walking AND training))) OR (TITLE-ABS-KEY (circuit AND training))) OR (TITLE-ABS-KEY (weight AND training))) OR (TITLE-ABS-KEY (chronic AND training))) OR (TITLE-ABS-KEY (lifestyle AND activity))) OR (TITLE-ABS-KEY (home AND training))) OR (TITLE-*

	ABS-KEY (water AND exercise))) OR (TITLE-ABS-KEY (tai chi AND training)))
**#2**	*((TITLE-ABS-KEY (liver AND steatosis)) OR (TITLE-ABS-KEY (non-alcoholic fatty liver AND disease)) OR (TITLE-ABS-KEY (NAFLD)) OR (TITLE-ABS-KEY (fatty liver disease)) OR (TITLE-ABS-KEY (hepatic)) OR (TITLE-ABS-KEY (NASH)) OR (TITLE-ABS-KEY (aminotransferase))) OR (TITLE-ABS-KEY (liver enzymes)) OR (TITLE-ABS-KEY (AST)) OR (TITLE-ABS-KEY (ALT)) OR (TITLE-ABS-KEY (FBG)) OR (TITLE-ABS-KEY (fating insulin)) OR (TITLE-ABS-KEY (HOMA-IR)) OR (TITLE-ABS-KEY (HBA1C))*
**#3**	*(TITLE-ABS-KEY (randomized AND controlled AND trial) OR TITLE-ABS-KEY (controlled AND clinical AND trial) OR TITLE-ABS-KEY (randomized AND controlled AND trials) OR TITLE-ABS-KEY (random AND allocation) OR TITLE-ABS-KEY (double AND blind AND method) OR TITLE-ABS-KEY (single AND blind AND method) OR TITLE-ABS-KEY (clinical AND trial) OR TITLE-ABS-KEY (clinical AND trials) OR TITLE-ABS-KEY (random))*
**#4**	***#1** AND **#2** AND **#3***
